# Molecular Characterization of Temozolomide-Treated and Non Temozolomide-Treated Glioblastoma Cells Released Extracellular Vesicles and Their Role in the Macrophage Response

**DOI:** 10.3390/ijms21218353

**Published:** 2020-11-07

**Authors:** Elisa Panzarini, Stefano Tacconi, Elisabetta Carata, Stefania Mariano, Ada Maria Tata, Luciana Dini

**Affiliations:** 1 Department of Biological and Environmental Sciences and Technologies (Di.S.Te.B.A.), University of Salento, 73100 Lecce, Italy; elisa.panzarini@unisalento.it (E.P.); stefano.tacconi@unisalento.it (S.T.); elisabetta.carata@unisalento.it (E.C.); stefania.mariano@unisalento.it (S.M.); 2 Department of Biology and Biotechnology “C. Darwin”, Sapienza University of Rome, 00185 Rome, Italy; adamaria.tata@uniroma1.it; 3 CNR Nanotec, Campus Ecotekne, University of Salento, 73100 Lecce, Italy

**Keywords:** glioblastoma, extracellular vesicles, pro-inflammatory macrophages, anti-inflammatory macrophages, temozolomide

## Abstract

Extracellular vesicles (EVs) are widely investigated in glioblastoma multiforme (GBM) for their involvement in regulating GBM pathobiology as well as for their use as potential biomarkers. EVs, through cell-to-cell communication, can deliver proteins, nucleic acids, and lipids that are able to reprogram tumor-associated macrophages (TAMs). This research is aimed to concentrate, characterize, and identify molecular markers of EVs subtypes released by temozolomide (TMZ)-treated and non TMZ-treated four diverse GBM cells. Morphology, size distribution, and quantity of small (sEVs) and large (lEVs) vesicles were analyzed by cryo-TEM. Quality and quantity of EVs surface markers were evaluated, having been obtained by Western blotting. GBM cells shed a large amount of EVs, showing a cell line dependent molecular profile A comparative analysis distinguished sEVs and lEVs released by temozolomide (TMZ)-treated and non TMZ-treated GBM cells on the basis of quantity, size and markers expression. Finally, the GBM-derived sEVs and lEVs, irrespective of TMZ treatment, when challenged with macrophages, modulated cell activation toward a tendentially M2b-like phenotype.

## 1. Introduction

Extracellular vesicles (EVs) are submicron structures released by all cell types into the extracellular microenvironment. EVs contain functional genomic (fragments of double stranded DNA, mRNAs, and non-coding RNAs), proteomic and lipidomic cargo transported from the host to recipient cells [1,2]. Two mechanisms of EVs biogenesis are known: direct budding of the plasma membrane and generation from late endosomes. The latter are formed by inward budding of the limited multivesicular body (MVB) membrane and then released into the extracellular space upon fusion of MVB containing endosomes with the plasma membrane. The exact nomenclature of EVs is still controversial, since no specific markers to clearly distinguish the biogenesis pathway are defined. In line with the recommendation stated by the International Society for Extracellular Vesicles (ISEV) in 2018, EVs can be named according to their size, with small EVs (diameter ˂ 200 nm) and large EVs (diameter >200 nm) [3].

EVs release can be constitutive or regulated by pathological conditions, such as cancer, immune responses, and cardiovascular, neurodegenerative, and metabolic diseases [4]. The set of carried molecules, reflecting the tissue of origin, could be an excellent reservoir of biomarkers allowing to discriminate between healthy cells and ill cells [5]. In cancer, this is particularly useful as the cargo can indicate the tumor stage, malignancy, progression, recurrence, and treatment validity [6]. Indeed, by participating in intercellular communication between cancer cells and stromal cells, EVs support several hallmarks of cancer, such as proliferation, escape from cell death, angiogenesis, invasion, and metastasis [7,8].

Recently, the relevance of EVs in Central Nervous System (CNS) cancers is the object of several studies, due to their ability to cross the Blood Brain Barrier (BBB) and circulate in peripheral blood [9]. Indeed, Glioblastoma Multiforme (GBM) accounts for 77% of malignant gliomas represents the second cause of cancer death in adults less than 35 years old, with a median survival of about 15 months and free survival before recurrence of GBM less than 24 weeks [10]. The poor prognosis of the GBM patients is linked to resistance of GBM cells to conventional therapies such as surgical resection, radiotherapy, and adjuvant chemotherapy with the alkylating agent temozolomide (TMZ).

EVs might play a key role in sustaining highly malignant gliomas, in tumor recurrence and as liquid biopsy biomarkers. It is known that the interaction between cancer and surrounding non cancer cells ensures tumor proliferation, brain invasion, angiogenesis, immunosuppression, and a favourable GBM microenvironment [11]. Tumor-associated macrophages (TAMs) are active and highly plastic immune cells and include two major ontogenetically different cell populations: (i) microglia and (ii) monocytes-derived macrophages (MDMs) [12]. TAMs recruited to the tumor bulk can be reprogramed by GBM cells and thus have a key role in the GBM cells ability to adapt and to survive therapeutic onslaught through suppression of adaptive immune responses [13]. In the cancerous tissues, two distinct phenotypes of macrophages can be expressed: classical macrophage activation or pro-inflammatory or M1, and alternative macrophage activation or anti-inflammatory or M2 [14]. In the GBM bulk, TAMs support the tumor acquiring a M2 phenotype that distinguishes GBM from lower grade gliomas [15].

The favourable microenvironment to sustain a tumor is maintained by the release of chemokines and cytokines. An additional form of communication in which signaling molecules are transferred from tumor cells to TAMs *via* EVs has been recently proposed [16]. Indeed, MDMs are recruited to the tumor site by molecules directly secreted or transported by EVs in the microenvironment of the GBM [11].

TMZ, that still represents the standard therapy of GBM together with surgery and/or radiotherapy, influences the release of GBM-derived EVs [17,18]. Characterization of GBM-derived EVs are gaining attention not only as a platform for liquid biopsy bypassing the use of conventional imaging techniques but also to understand the EVs role in the GBM microenvirons [16,19]. Proteomic, lipidomic, and genomic research of GBM have produced the molecular signature of GBM cells, even if still little information is available on the signature of U-87 MG cells, which represent the gold standard cells of IV grade glioma [20,21,22,23], as well as for TMZ-treated GBM cells whose molecular signature knowledge is at least poorly reported on [18,19,20,21,22,23,24]. Research is focused on the definition of the molecular signature of GBM released EVs, being considered keystones in the battle against GBM. Indeed, EVs could be useful in designing new therapies, as biomarkers for diagnosing and as non-invasive tool in monitoring GBMGBM status. Currently, proteomic studies are mainly directed to investigate EVs released in the blood of patients with different gliomas grade without discriminate EVs source or their specific origin, i.e., glioblastoma stem-like cells (GSCSs)-derived EVs [18,19,22]. However, these studies do not consider the eventual contribution of EVs subtypes (i.e., small or large EVs) in cancer progression. Keeping in mind the above considerations, one focus of our study has been the characterization of EVs, further concentrated in small (sEVs) and large (lEVs) vesicles, released by TMZ-treated and non TMZ-treated GBM cells. EVs have been characterized for size and quantity and for the molecular profile by detecting the presence of specific plasma membranes and cytosolic markers: Epidermal Growth Factor Receptor (EGFR), Epidermal Growth Factor Receptor variant III (EGFRvIII), Podoplanin (PDPN), Isocitrate dehydrogenase 1 (IDH1), and heat shock protein 25, 70, and 90 (Hsp25, Hsp70, Hsp90). These proteins, still little-studied in EVs, are known to play key roles in GBM biology. EGFR, also known as HER1 or ERBB1, is a transmembrane receptor of the ERBB family and its mutant form, EGFRvIII, occurs most commonly in GBM. Expression of EGFRvIII promotes cell proliferation, angiogenesis, and invasion [25]. PDPN is a cell surface membrane glycoprotein carrying a large number of O-glycosides [26]. PDPN is 26 and is indicator of malignancy and is related with GBM progression and invasion, and other cancers [27,28]. IDH1 is an enzyme that reversibly catalyzes isocitrate into α-KG accompanied with the generation of NADPH from NADP+ in cytosol. In primary GBM, IDH1 increases the intracellular levels of α-KG, NADPH, and lipid biosynthesis and decreases the ROS level [29] contributing to the aggressive clinical courses [30,31]. HSPs are evolutionary conserved molecular chaperones that perform broad and crucial roles in proteome homeostasis, an important process that preserves the integrity of proteins in health and disease. HSPs have been considered as possible targets for GBM therapy due their importance in different mechanisms that govern GBM malignance. HSP90 has been broadly studied as an important factor for GBM cell migration and invasiveness via activation of TLR-4; HSP70 confers resistance and prolongs the survival of GBM cells; Hsp 25, belonging to small HSPs with molecular weight ranging from 15 to 40 kDa, induces resistance to apoptosis [32].

Finally, after concentration and characterization of sEVs and lEVs secreted by TMZ-treated cells and non TMZ-treated cells, the main goal of the study has been the definition of the in vitro role played by sEVs and lEVs in directing the macrophages phenotype deciphering the promotion of a pro or anti-inflammatory microenvironment.

## 2. Results

### 2.1. Cryo Electron Microscopy Characterization and Molecular Profile of EVs from GBM Cells

The EVs released by four GBM cell types (U-87 MG, U-373 MG, T 98G, and U-251 MG) concentrated by differential ultracentrifugation of the conditioned media (CM) were characterized by cryo-transmission electron microscopy (cryo-TEM). Although differential ultracentrifugation does not provide full separation of the different types of EVs, it is the current common method of EVs preparation [33]. Hence, for the purposes of this publication, we will refer to the 20,000 g pellet as large vesicles, lEVs (diameter > 200 nm), and to the 100,000 g pellet as small vesicles, sEVs (diameter ˂ 200 nm).

**Figure 1:**
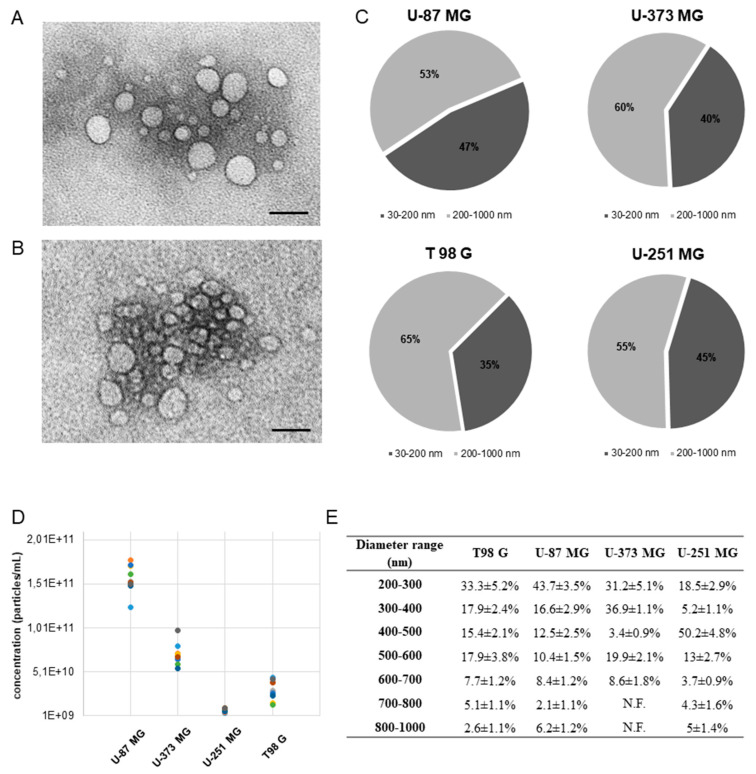
Characterization of EVs concentrated from U-87 MG, U-373 MG, T 98G, and U-251 MG cells. (**A**) Cryo-transmission electron microscopy (cryo-TEM) images of U-251 MG cells derived EVs. (**B**) Cryo-transmission electron microscopy (cryo-TEM) images of U-87 MG derived EVs. Bar = 300 nm. (**C**) Size distribution of EVs released by GBM cells evaluated by cryo-TEM. EVs are categorized as small (range diameter 30–200 nm) and large (range diameter 200–1000 nm) vesicles. The percentage of sEVs and lEVs sub-types was obtained by measuring the diameter of almost 500 vesicles for each sample. The colored dots correspond to each repeated experiment for each sample. (**D**) Concentration (particles/mL) of EVs in culture medium of GBM cells. Data reported correspond to *n* = 3 samples in 3 independent experiments. (**E**) Size distribution of large EVs released by GBM cells evaluated by cryo-TEM. The percentage of each EVs group was obtained by measuring the diameter of almost 500 vesicles for each sample. Data reported correspond to *n* = 3 samples in 3 independent experiments. N.F.: Not Found.

EVs morphology, size distribution, and quantity (particles/mL) data were obtained with cryo-TEM. Direct cryo-TEM imaging allowed us to obtain the near-to-naïve nano-morphology of EVs. All the vesicles were round-shaped without variation relative to the cells of origin. Cryo-TEM images of sEVs and lEVs of U-87 MG (Figure 1A) and U-251 MG (Figure 1B) cells are reported. The EVs quantity and ratio between sEVs and lEVs changed depending on the GBM cell lines considered (Figure 1C). lEVs were more numerous than sEVs regardless of cell type (Figure 1C). U-87 MG cells produced the highest quantity of particles/mL followed by U-373 MG, T98 G, and U-251 MG (Figure 1D). The average size and the size distribution of sEVs were quite homogeneous, with an average diameter of 85 ± 1.2 nm and a range diameter of 35–150 nm. The size of lEVs was more heterogeneous, with an average diameter swinging from the smallest 282 ± 70 nm of U-87 MG to the greatest 430 ± 168 nm of U-251 MG. Range diameters of lEVs are reported in Figure 1E.

In Table 1 and Supplementary Figure S1, the molecular markers (EGFR, EGFRvIII, PDPN, IDH1, Hsp25, Hsp70, and Hsp90) detected on sEVs and lEVs of the four GBM cells types are reported. A particularly heterogenous presence of markers among the different GBM cells and between sEVs and lEVs of the same origin cell was observed. The simultaneous presence of all markers (with the unique exception of PDPN on sEVs) was observed only for the U-87 MG cell-derived EVs (Table 1, Supplementary Figure S1). The further validation of sEVs and lEVs has been performed by evaluating the presence of specific markers: CD63 for sEVs and Annexin A1 for lEVs (Supplementary Figure S2).

### 2.2. Cell Surface Distribution and Intracellular Localization of Small and Large Vesicles on GBM Cells

**Figure 2:**
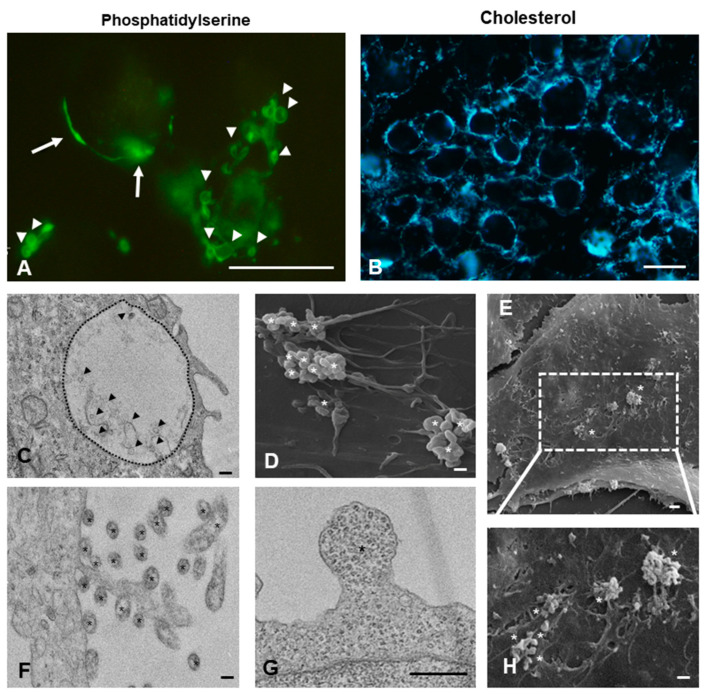
Fluorescent and electron microscopic analysis of U87-MG cells. (**A,B**) Fluorescent labeling of phosphatidylserine residues (FITC-conjugated annexin-V) and cholesterol (filipin) on plasma membrane of U-87 MG cells. Arrow: clustering of PS residues. Arrow head: vesicles. Bar = 10 µm. Transmission Electron Microscopy (TEM) (**C,F,G**) and Scanning Electron Microscopy (SEM) of U-87 MG (**D,E,H**) cells. Micrographs show large EVs (asterisks) shed from plasma membrane and small EVs (arrow heads) inside multivesicular bodies (dashed black line). Bar = 200 nm.

Phosphatidylserine residues and cholesterol, described to be associated to plasma membrane rafts, are pivotal for budding and consequent vesiculation of lEVs. The surface of GBM cells was rich in both of these molecules (Figure 2A,B). GBM cells actively secret sEVs and lEVs, whose intracellular localization and cell surface distribution, as observed by TEM and SEM, are shown in Figure 2C-H. Examples of lEVs budding from the plasma membrane (Figure 2G and Supplementary Figure S3G) and lEVs still attached or just detached from cell (Figure 2F and Supplementary Figure S3H) are given. The cell surface distribution of lEVs derived from U87 MG cells, better observed with SEM, is as clusters preferentially localized on filopodia (Figure 2D,E,H). On the other hand, on U-251 MG and T98 G cells, lEVs are mainly observed on the lamellipodia organized in small clusters (Supplementary Figure S3A,B,E). sEVs were always found within the multivesicular bodies (Figure 2C and Supplementary Figure S3C,D,F,H).

### 2.3. Sensititvity to TMZ Treatment of GBM Cells

The four GBM cell types, treated with increasing concentration of TMZ (from 50 to 400 µM) for 24, 48, and 72 h, were not equally responsive to TMZ treatment. U-87 MG cells were the most sensitive cells to TMZ treatment (decrease in cell viability of about 75%) and U-373 MG the most resistant (decrease in cell viability of about 40%) (Supplementary Figure S4). The most efficient TMZ concentration and incubation time in U-87 MG viability reduction were, respectively, 200 µM and 48 h.

### 2.4. EVs Signature

**Figure 3:**
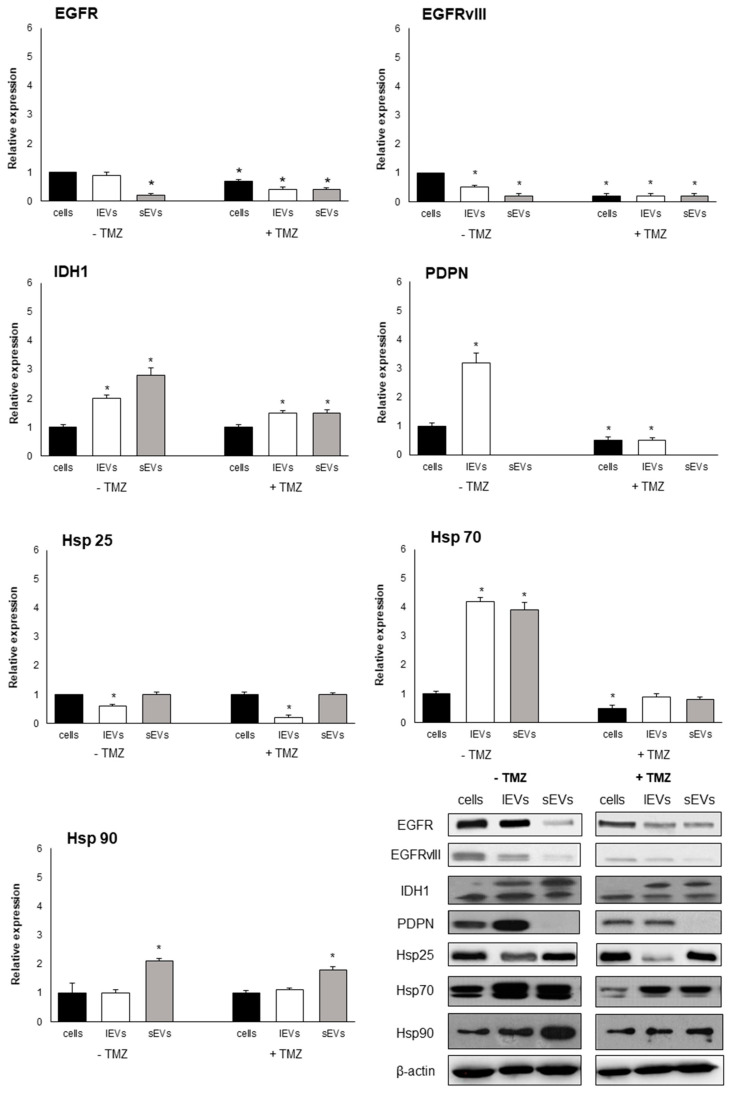
The expression levels of epidermal growth factor receptor (EGFR), EGFRvIII, isocitrate dehydrogenase 1 (IDH1), podoplanin (PDPN), Hsp25, Hsp70, and Hsp90 in U-87 MG cells and in isolated lEVs and sEVs after 200 µM temozolomide (TMZ) for 48 h treatment evaluated by Western blotting analysis. The expression of EGFR, EGFRvIII, IDH1, and PDPN is normalized with the housekeeping protein (β-actin) and reported as relative expression respect to parental cells. The values for all experimental groups reported in the histograms represent the means ± SD (*n* = 3) of three independent experiments; (*) *p* < 0.05 compared to respective origin cells. Representative images of proteins blots are reported.

To determine whether molecular markers could help to differentiate EVs derived from TMZ-treated cells and from non TMZ-treated cells, the expression of EGFR, EGFRvIII, IDH1, PDPN, Hsp25, Hsp70, and Hsp90 proteins in EVs-derived from 200 µM TMZ treated and untreated U-87 MG cells were analyzed and compared with origin cells and with the non TMZ-treated cells. Regardless of the TMZ treatment, EVs markers are qualitatively similar but quantitatively different when compared to the origin cells (Figure 3). A general significative decrease in the expression of EVs markers was observed in TMZ treated U-87 MG cells with respect to non-TMZ-treated U-87 MG cells (Figure 3 and Supplementary Figure S5A). Indeed, for example, PDPN and IDH1 PDPN and IDH1markers show a strong decrement when produced by TMZ-treated GBM. In general, the expression of some markers was different between sEVs and lEVs. Interestingly, by comparing the differences between large and small vesicles of the same treatment (+TMZ or -TMZ), only the differences measured in the non TMZ treated U87MG were significant (Figure 3 and Supplementary Figure S5A).

### 2.5. Effects of U-87 MG-Derived EVs on Macrophage Polarization

**Figure 4:**
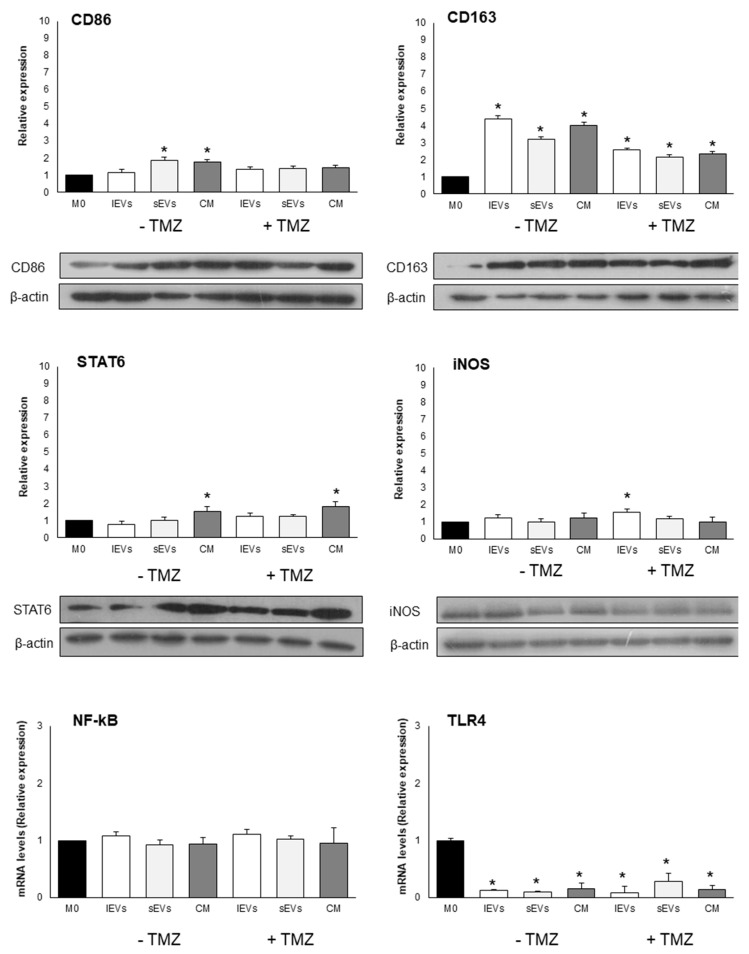
Macrophage polarization markers after exposure to 200 µM temozolomide (TMZ) for 48 h treated or non-TMZ- treated U-87 MG cells derived lEVs, sEVs and free EVs CM for 24 h. The expression of CD86, CD163, STAT6 and iNOS is normalized with the housekeeping protein (β-actin) and reported as relative expression respect to control M0. The mRNA levels of TLR4 and NF-kB are normalized to GAPDH mRNA level and expressed as relative expression respect to M0. The values for all experimental groups reported in the histograms represent the means ± SD (n = 3) of three independent experiments; (*) *p* < 0.05 compared to control M0. Representative images of proteins blots are reported.

**Figure 5:**
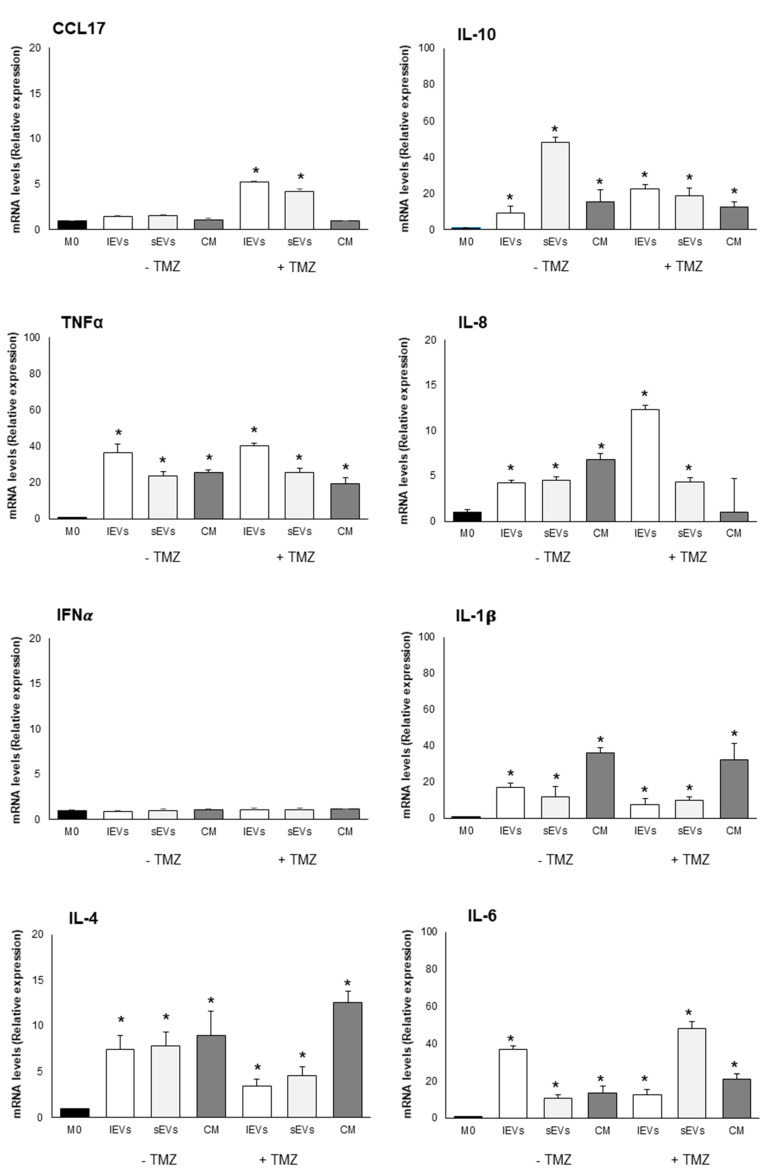
Macrophage polarization markers after exposure to 200 µM temozolomide (TMZ) for 48 h treated or non TMZ- treated U-87 MG cells derived lEVs, sEVs and free EVs CM for 24 h. The mRNA levels of CCL17, IL-10, TNFα, IL-8, IFNα, IL-1β, IL-4, and IL-6 are normalized to GAPDH mRNA level and expressed as a relative expression with respect to M0. The values for all experimental groups reported in the histograms represent the means ± SD (n = 3) of three independent experiments; (*) *p* < 0.05 compared to the control M0.

To investigate the role of GBM-derived EVs in reprogramming macrophage phenotype and functions, TPA differentiated THP-1 cells (M0 macrophages) were cultured for 24 h in the presence of sEVs and lEVs isolated by culture medium of TMZ-treated and non-TMZ-treated U-87 MG cells. In Figure 4 and Figure 5, in Table 2, and in Supplementary Figure S5B are reported the expression levels of M0 macrophages polarization markers after challenging with the diverse EVs. Quantitative differences, which in some cases were of considerable significance, were found to be dependent on the EVs subtype and treatments. Of note is the fact that IFNα expression never changed and the level of TLR4 dramatically decreased irrespective of EVs subtypes added and TMZ treatment if compared to the control, non TMZ-treated M0 macrophages without the addition of EVs. An amount of 200 µM TMZ alone and the vehicle in which TMZ is dissolved, i.e., DMSO, did not affect the viability of M0 macrophages (Supplementary Figure S6). The effects on macrophages polarization were not observed after the treatment of M0 macrophages with resulting EVs-free conditioned medium from TMZ-treated and untreated U-87 MG cells, thus excluding a possible role of soluble molecules.

## 3. Discussion

In the present study, pieces of evidence highlight the role of GBM released EVs in modulating macrophage phenotype and microenvironment inducing an M2b-like polarization that sustains an immunosuppressive environment. Data on the potential role of EVs to discriminate between non TMZ-treated GBM and TMZ-treated GBM cells were also reported.

We preliminarily analyzed four different GBM cells (U-373 MG, U-251 MG, T 98G, and U-87 MG) to establish the better in vitro model in term of releasing high EVs yields and high susceptibility to TMZ. U-87 MG cells met these characteristics and were chosen as first experimental cell model to be investigated, being well aware that this is only a GBM subtype and that it is not representative of the entire GBMs; thus, further experiments with other GBM cell lines are needed.

Despite the fact that human tumor-derived cell lines are indispensable tools for basic and translational oncology, for their infinite life span, easy handling, and high reproducibility of results, and that U-87 MG represents the gold standard cells of IV grade glioma, it should be taken into account that a tumor-derived cell line may not be authentic to the tumor of origin. Indeed, the DNA profile of the widely used glioma cell line U-87 MG is different from that of the original cells and thus U-87 MG is a human glioblastoma cell line of unknown origin [34]. In line with the important burst received by EVs research in recent years, we focused our research on the TMZ-treated and non TMZ-treated U-87-GM-derived EVs. The idea that EVs, released by most cells, were just cell disposal routes without biological significance has dramatically changed with the recognition that EVs contain biologically active cargo that the cells send to local or distant targets to modulate specific biological functions. The dimensionality of the EVs research field has expanded with the recognition of diverse sub-types of EVs and the complexity of vesicle biogenesis, cargo loading, release pathways, targeting mechanisms, and vesicle processing.

Despite the fact that there are disputes in biogenesis differentiation, i.e., direct shedding of plasma membrane or release after fusion of multivesicular body with plasma membrane, as well as in the specific difference between the sEVs and lEVs, due to the presence in all EVs of plasma membrane and cytosolic proteins [35], we analyzed sEVs and lEVs separately. Indeed, we not only isolated the GBM-derived EVs, but we separated EVs on the basis of the size in sEVs and lEVs before their characterization in terms of quantity, size distribution, and molecular profile. To our knowledge, this is the first work that analyzes GBM-derived sEVs and lEVs separately in all experiments.

Our data indicate that all four GBM cells shed an average high number of EVs. The molecular profile of EVs was cell line dependent, reflected the GBM origin cell, and, finally, differences between the sEVs and lEVs were observed in terms of size, concentration, and molecular profile. In line with Lane et al., these data reported that sEVs content mirrors the phenotypic signature of the respective GBM cells, leading to the description of potential sEV-associated biomarkers for GBM subtyping [20]. A detailed knowledge of the different molecular signature of the GBM cell-derived EVs is helpful in the characterization of distinct subtypes or, for example, GBM resistance to therapy [22,36,37]. Proteomic analysis of pro-neural and mesenchymal GBM stem cell-produced EVs revealed pro-angiogenic and invasiveness molecules [22,36]. The molecules characterizing GBM cell-released EVs would be of great value in the search for potential biomarkers of disease. Indeed, EVs are considered an ideal tool to achieve information about the GBM that, currently, are ensured only by biopsy. In 2012 was published the first paper about the possibility of performing liquid biopsy in GBM patients [19]. EVs-based liquid biopsy could have some advantages compared with conventional biopsy: it is rapid, simple, less invasive in its diagnostic method, and efficient in monitoring the efficacy of the treatment [38]. EVs molecules are modulated by TMZ treatment, thus allowing us to discriminate between the non TMZ-treated and TMZ- treated GBM cells. For example, abundant levels of cell adhesion-related proteins, including cell adhesion molecules (CAMs) and β1-integrin, were detected in EVs released by TMZ-treated glioblastoma stem-like cells obtained from primary glioblastoma resection [18]. Indeed, differences were also found in the EVs subtypes. U87 MG are very sensitive to the TMZ treatment and underwent a massive cell death. We found that the number of released EVs was influenced by TMZ and that the cargo of vesicles in terms of both type of molecules and amount was affected as well. That TMZ treatment interferes with the number of released vesicles is still controversial in literature as some papers report an increase in the amounts of released vesicles, and others report a decrease [18,19]. However, before each testing of the presence of cargo molecules (inside and on the membrane of EVs from U87 MG treated or not with TMZ) the vesicles integrity was checked by cryo-TEM.

The response of the cells to a treatment implies an alteration of the cellular metabolic activity that reflects the type and the amounts of molecules. This naturally occurring response of the cells also reflects the type and amounts of molecules present into the extracellular vesicles. Our experiments demonstrated high levels of IDH1, PDPN, Hsp70, and Hsp90 in sEVs and lEVs, suggesting that these proteins sustain the GBM growth and invasiveness and could be exploited as new biomarkers of the disease. Indeed, the PDPN potential to be used as a biomarker in liquid biopsy was already suggested [19]. Here, PDPN and Hsp70 in EVs encourage the use of a combination of molecular markers, and, thus, we hypothesize that these proteins could be considered as GBM biomarkers. Since levels of PDPN, Hsp70, IDH1, and EGFR and its variant form decreased in EVs from TMZ-treated GBM cells, their use could also indicate the effectiveness or otherwise of the therapy. Future investigations should focus on the surface or lumen localization of these EVs markers, to better understand their role in GBM biology.

The EVs, with diameters ranged about from 30 to 800 nm, were almost equally divided between sEVs and lEVs (47% vs. 53%, respectively)) in all experimental conditions. The mean diameter of sEVs was 85 nm and about 50% of lEVs has diameter in the range 200–300 nm, in line with the sizes reported in literature [3]

Beyond the mere morphology, the EM imaging confirms the release of EVs by GBM cells and the high production of EVs in U-87 MG cells. By combining SEM and TEM imaging, the presence, amount and, in particular, distribution of vesicles on cell surface, was visualized. Of particular interest is the observation that most of the lEVs are formed and released in a specific cellular area and that differences of EVs distribution in non TMZ-treated and TMZ-treated cells were observed. For example, the large clusters of lEVsof lEVs were distributed on the U-87 GM filopodia, while in the UU-251 MG and T98 G cells lEVs concentrated as small clusters on the lamellipodia.

Since the reason for the budding of lEVs or secretion of MVB in a specific area of the plasma membrane is still unknown, the data showing different distribution and clustering of EVs could be an important point for further investigations of how and why EVs are released in a well-defined part of the cells. It is possible that determined intracellular pathways could drive toward a specific destination on the cell surface where are concentrated the molecules with which to enrich the surface of the EVs.

To verify our hypothesis regarding the presence of different intracellular pathways and signaling for the formation of large vesicles, further investigation is needed. Extensive labeling of phosphatidylserine and cholesterol is in line with what above discussed.

After the characterization of sEVs and lEVs, we investigated in vitro, for the first time to our knowledge, the macrophage phenotype modulation driven by the EVs subtypes. In this work, we (1) demonstrated the effect of EVs released by TMZ- treated and non-TMZ-treated GBM cells on macrophages activation, (2) investigated the contribution to macrophages activation of sEVs and lEVs, and (3) investigated the contribution to macrophages activation of soluble molecules present in the conditioned medium of TMZ treated and non TMZ treated GBM cells.

The results obtained demonstrated that TMZ-treated and non-TMZ-treated GBM cells derived EVs induced in vitro a M2b-like phenotype that in vivo may favour tumor recurrence and progression. Interestingly, we found that anti and pro-inflammatory molecules were expressed, suggesting a complex network of signaling. In fact, high levels of TNFα and IL-1β (pro-inflammatory cytokines), IL-10 (anti-inflammatory cytokines), and Il-6 (pro- and anti-inflammatory cytokine in cancer) were detected and suggest that macrophages tendentially acquire a M2b activation, that possess both protective and pathogenic roles in cancer [39]. The polarization to M2-like phenotype was described for monocytes internalizing GSCs-derived exosomes [40]. Evidence from the last few years shows that EVs released by GBM cells support macrophages phenotype modulation toward a M2-like phenotype leading to immunosuppression, angiogenesis, and tumor promotion [41,42,43]. In fact, de Vrij et al. [44] reported that EVs released by U-87 MG cells and GBM primary cultures induced monocytes and microglia toward a M2-like anti-inflammatory phenotype and that activated macrophages to M2-like phenotype who showed an increased phagocytic capacity. Again, EVs released by GL261 cells and U-87 MG and GSCs [40,45] mainly target monocytes isolated from blood to acquire a M2-like phenotype.

Soluble molecules released in the CM resulting after EVs concentration contributed to modulate GBM microenvironment. [46]. A role of CM of GBM cells TMZ-treated and non TMZ treated to modulate macrophages activity has been already demonstrated [46]. However, in our hands, soluble molecules induced macrophages to produce fewer anti-inflammatory cytokines than pro-inflammatory cytokines. It is not completely clear if these cytokines are released by macrophages or are the result of some signaling released by damaged vesicles.

Indeed, our results show a modulation of the macrophages activation status toward a M2b-like polarization and a partial immunosuppressive microenvironment.

## 4. Materials and Methods

### 4.1. Cell Culture and TMZ Treatment

U-87 MG, U-373 MG, T98 G, and U-251 MG human GBM cell lines were cultured in Eagle’s Minimum Essential Medium (EMEM) (Cambrex, Verviers, Belgium) supplemented with 10% of heat inactivated Fetal Bovine Serum (FBS) (Cambrex, Verviers, Belgium), 2 mM L-glutamine (Cambrex, Verviers, Belgium), 100 IU/mL penicillin and streptomycin solution and 10,000 U/mL amphotericin (antimicotic solution) (Sigma-Aldrich, St. Louis, MO, USA) in a 5% CO_2_ humidified atmosphere at 37 °C. The cell lines were purchased by Cell bank IRCCS AOU San Martino IST (Genova, Italy). Cells were maintained in 75 cm^2^ flasks (concentration ranged between 2 × 10^5^ and 1 × 10^6^ cells/mL) by passage every 3 to 4 days. From the temozolomide (TMZ, Selleckchem.com, Munich, Germany) stock solution (100 mM in dimethyl sulfoxide, DMSO obtained by solubilizing 10mg of TMZ in 0.515 mL of DMSO), 50, 100, 200, and 400 µM TMZ were added to 15 × 10^4^/well cells for 24, 48, and 72 h, to assay the most effective conditions, the most sensitive cells and the effects on EVs release. Human THP-1 acute monocytic leukemia cells were cultured in RPMI-1640 medium (Cambrex, Verviers, Belgium) supplemented with 10% of heat inactivated Fetal Bovine Serum (FBS) (Cambrex, Verviers, Belgium), 2 mM L-glutamine (Cambrex, Verviers, Belgium), 100 IU/mL penicillin and streptomycin solution and 10,000 U/mL amphotericin (antimicotic solution) (Sigma-Aldrich, St. Louis, MO) in a 5% CO_2_ humidified atmosphere at 37 °C. The cells were maintained in 75 cm^2^ flasks at a concentration of 5 × 10^5^ cells/mL by passage every 3 to 4 days. THP-1 monocytes were differentiated into macrophages (M0) by 72 h incubation with 100 ng/mL Phorbol 12-Myristate 13-Acetate (PMA) as previously reported [47,48].

Before each treatment, complete culture medium was centrifuged at 100,000× *g* at 4 °C overnight to remove EVs from FBS (Supplementary Figure S7).

### 4.2. Fluorescence Microscopy

The presence and distribution of phosphatidylserine and cholesterol residues on GBM cells plasma membrane were analyzed with a fluorescence microscope Eclipse 80i (Nikon, Kawasaki, Kanagawa Prefecture, Japan). Phosphatidylserine residues were labelled for 10 min at 37 °C with 0.5 mg/mL of FITC-conjugated annexin-V (#A9210; Sigma-Aldrich, St. Louis, MO, USA) in the complete culture medium of cells grown on coverslips (1 × 10^5^ cells/13 mm diameter coverslip) after two extensive rinsings with 0.2M of PBS (pH 7.4).

Cholesterol was labeled for 2 h at 37 °C with a 0.05-mg filipin (#70440; Cayman Chemical, Milan, Italy)/mL of PBS (0.2 M), pH 7.4, in complete culture medium of cells grown on coverslips (1 × 10^5^ cells/13 mm diameter coverslip) after two extensive rinsing with 0.2M of PBS (pH 7.4).

Before fluorescence microscopy, observation slides were extensively rinsed with 0.2M of PBS (pH 7.4) and glycerine mounted medium.

### 4.3. Electron Microscopy

GBM cells, cultured on glass coverslips (1 × 10^5^ cells/13 mm diameter coverslip), were fixed with 2.5% glutaraldehyde in 0.1 mol/L cacodylate buffer pH 7.4 for one hour at ice temperature and post-fixed with 1% OsO_4_ in the same buffer for 1 h on melting ice. After fixation, cells were dehydrated with increasing degrees of acetone (25%, 50%, 70%, 90% and 100%) followed by critical point drying (Critical Point Dryer CPD EMITECH K850; Quorum Technologies Ltd., Laughton East Sussex, UK). Stub-mounted specimens were gold-coated using a Balzers Union SCD 040 (Balzers Union, Liechtenstein) and examined under a Zeiss EVO HD 15 (Carl Ziess, Oberkochen, Germany) scanning electron microscopy (SEM).

GBM cells were fixed with 2.5% glutaraldehyde in 0.1 mol/L cacodylate buffer pH 7.4 for 1 h at ice temperature and post-fixed with 1% OsO_4_ in the same buffer for 2 h at ice temperature. Cells were then dehydrated with increasing degrees of ethanol (25%, 50%, 70%, 90%, and 100%) and embedded in Epoxy Spurr resin (# 4221D-1; TAAB Laboratories Equipment Ltd., Aldermaston, Berks, RG7 8NA, England). The 60 nm sections uranyl acetate and lead citrate stained were examined under a Hitachi 7700 (Hitachi High Technologies America Inc., Dallas, TX) transmission electron microscope (TEM) operating at 100 kV.

### 4.4. Isolation and Characterization of EVs

After TMZ and non TMZ treatments of GBM cells, EVs were isolated from the culture medium by differential centrifugation using a Beckman Coulter Ultracentrifuge Optima XE (Beckman Coulter, Brea, CA, USA). The culture medium was first centrifuged at 500× *g* for 10 min at room temperature, and the resulting supernatant was then centrifuged at 800× *g* for 10 min at room temperature. The last centrifugation at 2000× *g* for 20 min at room temperature of the supernatant was done to remove cells or cells aggregates. Cell-depleted supernatant was centrifuged at 20,000× *g* for 20 min at 4 °C, and then the pellet containing large vesicles (lEVs) was collected. The supernatant was filtered through a 0.22 μm filter (filter units with polyethersulfone (PES) membrane, Thermo Fisher Scientific, Waltham, MA, USA) and then centrifuged at 100,000× *g* for 70 min at 4 °C and the pellet containing small vesicles (sEVs) was collected. The supernatant was taken as EVs free conditioned medium (CM).

The size and number of EVs were analyzed by Cryo-Transmission Electron Microscopy (cryo-TEM). 3–5 µL of each EVs sample suspended in PBS were put on a carbon-coated grid. The grid was maintained at 85% humidity for 10 s and then plunge-frozen in liquid ethane using a Vitrobot IV Mark (FEI, Hillsboro, OR, USA). The grids placed on a cryo-transfer specimen holder (Gatan, Pleasanton, CA, USA), at −175 °C, were observed with a cryo-TEM Hitachi 7700 (Hitachi High Technologies America Inc., Dallas, TX, USA) operating at 100 kV.

sEVs and lEVs were analyzed for the protein expression of epidermal growth factor receptor (EGFR), epidermal growth factor receptor variant III (EGFRvIII), isocitrate dehydrogenase 1 (IDH1), podoplanin (PDPN), heat shock proteins 25, 70, and 90 (Hsp25, Hsp70, Hsp90) by Western Blot as detailed below.

### 4.5. Co-Culture Experiments

The 2 × 10^10^ sEVs or lEVs were added to 1 × 10^6^ M0 macrophages in RPMI-1640 medium. M0 macrophages were cultured also in EVs free conditioned medium (CM) from U-87 MG GBM cells to assess the effects of released soluble molecules. After incubation for 24 h, cells were prepared for Western Blotting and RT-PCR analysis.

The polarization markers analyzed are: (a) M1 macrophage markers: inducible nitric oxide synthase (iNOS), cluster of differentiation 86 (CD86), Toll-like receptor 4 (TLR4), subunit p65 of nuclear factor kappa-light-chain-enhancer of activated B cells (NFkB-p65), tumor necrosis factor α (TNFα), interleukin 6 (IL-6), interleukin 8 (IL-8), interleukin 1β (IL-1β), interferon γ (IFNγ); (b) M2 macrophage markers: cluster of differentiation 163 (CD163), signal transducer and activator of transcription 6 (STAT6), interleukin 4 (IL-4), interleukin 10 (IL-10), and C-C motif chemokine ligand 17 (CCL17).

### 4.6. Western Blotting

GBM cells (1 × 10^6^) or EVs (2 × 10^10^) or M0 macrophages (1 × 10^6^) were lysed in radioimmunoprecipitation assay (RIPA) buffer (NaCl 150 mM, Tris-HCl 50 mM pH 8, MgCl_2_ 2 mM, SDS 0.1%, Deoxycholic Acid 0.5%, NP40 1%) containing phenylmenthylsulfonyl fluoride (PMSF) and protease inhibitor cocktail (ThermoFisher Scientific, Waltham, MA). After sonication (5 min), insoluble material was pelleted by centrifugation for 30 min at 13,000× *g* at 4 °C. Supernatants were transferred to a new tube, and the protein concentration was measured by Bradford assay. Proteins (10µg) were separated by sodium dodecylsulfate-polyacrylamide gel electrophoresis (10–12% polyacrylamide, SureCast™ Acrylamide Solution, ThermoFisher Scientific, Waltham, MA, USA) and transferred to nitrocellulose membranes. The membranes were blocked with 5% nonfat dry milk in Tris-buffered saline containing 0.1% Tween 20 (TTBS) for 1h at room temperature. Then, the membranes were incubated overnight at 4 °C with following primary antibodies: EGFR (1:1000 diluition; #sc-120, Santa Cruz biotechnology Inc, Dallas, TX, USA) EGFRvIII (1:1000 diluition; #sc-373746, Santa Cruz biotechnology Inc, Dallas, TX, USA.) IDH1 (1:1000 diluition; #sc-515396, Santa Cruz biotechnology Inc, Dallas, TX, USA) PDPN (1:500 diluition; #sc-376695, Santa Cruz biotechnology Inc, Dallas, TX, USA) Hsp25 (1:500 diluition #H1154, Sigma-Aldrich, St. Louis, MO, USA), Hsp70 (1:2000 diluition #SAB1301520, Sigma-Aldrich, St. Louis, MO, USA), Hsp90 (1:400 diluition #SRP5191, Sigma-Aldrich, St. Louis, MO, USA); anti-iNOS (1:4000 dilution; #PA3-030A, Merck Millipore, Darmstadt, Germany), anti-CD163 (1:1000 dilution; #PA5-109327, ThermoFisher Scientific, Waltham, MA, USA), anti-CD86 (1:1000 dilution; #MA5-15697, ThermoFisher Scientific, Waltham, MA, USA), anti-STAT6 (1:1000 dilution; #9362, Cell Signaling Technology, Danvers, MA, USA) and anti-CD63 (1:1000 dilution; #ab216130, Abcam, Cambridge, MA, USA) and anti-Annexin A1 (1:1000 dilution; #ab214486, Abcam, Cambridge, MA, USA) diluted in TTBS in 5% non-fat dry milk. Anti-human β-actin (1:2000 dilution; #A1978 Sigma-Aldrich, St. Louis, MO, USA) in TTBS in 5% non-fat dry milk was used as control. After washing with TTBS, the membranes were incubated for 1 h at room temperature with the following horseradish peroxidase-conjugated secondary antibody diluted in TTBS in 5% non-fat dry milk: rabbit anti-mouse IgG (1:5000 dilution; #A9044, Sigma-Aldrich, St. Louis, MO, USA), goat anti-rabbit IgG (1:5000 dilution; #A0545, Sigma-Aldrich, St. Louis, MO, USA). The immunoreactive bands were detected by ChemiDoc Imaging System (Bio-Rad Laboratories, Hercules, CA, USA) using a commercial enhanced chemiluminescence (ECL) reagent (Immobilon Crescendo Western HRP substrate; Merck Millipore, Darmstadt, Germany). The density of the specific bands was quantified by densitometric analysis performed by Image Lab SoftwareTM.

### 4.7. RNA Extraction, cDNA Synthesis and Real-Time PCR

Total RNA was obtained from macrophages according to user manual of Trizol (Invitrogen, Carlsbad, CA, USA). After isolation, the concentration of RNA was determined by Nanodrop ND-1000 Spectrophotometer (Thermo Fisher Scientific, Waltham, MA, USA). 1.5 µg of RNA was converted to cDNA using a single-step SuperScriptTM IV kit (Invitrogen, Carlsbad, CA, USA) protocol. Each reaction was performed in triplicate, using mRNA levels of Glyceraldehyde 3-phosphate dehydrogenase (GADPH) for normalization. Quantitative gene expression analysis was performed using CFX ConnectTM Real-Time PCR Detection System (BIORAD, Hercules, CA, USA).

The sequences of the primers used for Real-Time PCR analysis were as follows:

IL-1β (forward 5′-GTGTTCTCCATGTCCTTTGT-3′; reverse 5′- CATATGGACCAGACATCAC -3′); IL-6 (forward 5′-CAATCTGGATTCAATGAGGAGAC-3′; reverse 5′-CTCTGGCTTGTTCCTCACTACTC-3′); IL-10 (forward 5′-GATGCCTTCAGCAGAGTGAA-3′; reverse 5′- GCAACCCAGGTAACCCTTAAA-3′); IFNα (forward 5′-GTGAGGAAATACTTCCAAAGAATCAC-3′; reverse 5′-TCTCATGATTTCTGCTCTGACAA-3′); TNFα (forward 5′-GCCCATGTTGTAGCAAACCCTCAA-3′; reverse 5′-TGGCACCACCAACTGGTTATCTCT-3′); IFNγ (forward 5′-TGACCAGAGCATCCAAAAGA-3′; reverse 5′-CTCTTCGACCTCGAAACAGC-3′); CCL17 (forward 5′-CTTCTCTGCAGCACATCCA-3′; reverse 5′-GTACCACGTCTTCAGCTTTCT-3′); TLR4 (forward 5′- TATCAGAGCCTAAGCCACC -3′; reverse 5′- GTCTCCACAGCCACCAGCT -3′); NF-kB-p65 (forward 5′-CTGAACCAGGGCATACCTGT-3′; reverse 5′-GAGAAGTCCATGTCCGCAAT-3′); GAPDH (forward 5′-CGCTTCGCTCTCTGCTCCT-3′; reverse 5′-CCGTTGACTCCGACCTTCAC-3′).

### 4.8. Statistical Analysis

All data are expressed as mean ± SD. Multiple comparisons were performed by two-way ANOVA. Comparisons between two groups were performed using a Student’s t-test (GraphPad Prism 7 software, GraphPad Software, San Diego, CA, USA), and *p* < 0.05 was considered to be significant.

## 5. Conclusions

The present work describes the characterization of sEVs and lEVs released from different GBM cell lines. Moreover, for the first time, it highlights the different involvement of sEVs and lEVs released by TMZ- treated and non TMZ-treated U-87 MG cells in modulating activation of macrophages, opening new perspectives in tumor immunology. Indeed, our results show a modulation of the macrophages activation status toward M2b-like polarization and a partial immunosuppressive microenvironment. The M2b-like phenotype acquired by macrophages, may result enable to develop an efficient immunotherapy against GBM.

Finally, we identified on EVs some molecules, like PDPN and Hsp70, that could be exploited as useful markers in understanding efficacy of TMZ treatment.

In conclusion, the present study, albeit requiring further investigation extensible to other GBM cell lines as well as in primary GBM cells, confirm the cross-talk between GBM cells and macrophages in the modulation of the microenvironment favourable to tumor cells survival but highlights also the potential use of the EVs markers as possible tools to evaluate the efficacy of the chemotherapy drugs treatment.

## Figures and Tables

**Table 1:** Expression of markers in T98 G, U-87 MG, U-373 MG, and U-251 MG derived large and small EVs.

Molecules	Large Vesicles				Small Vesicles			
	T98G	U87MG	U373MG	U251MG	T98G	U87MG	U373MG	U251MG
PDPN		+						
EGFRvIII	+	+	+	+		+		
EGFR		+				+		
IDH1		+				+		
Hsp70		+				+		+
Hsp90		+		+		+	+	
Hsp25		+				+		

EGFR: epidermal growth factor receptor, EGFRvIII: epidermal growth factor receptor variant III, IDH1: isocitrate dehydrogenase 1, PDPN: podoplanin, Hsp25: heat shock protein 25, Hsp70: heat shock protein 70; Hsp90: heat shock protein 90. (+): presence.

**Table 2:** Macrophage polarization markers expression following incubation with small and large EVs or CM of TMZ-treated U87 MG cells and non TMZ-treated U87 MG cells.

		-TMZ			+TMZ		
		Large EVs	Small EVs	CM	Large EVs	Small EVs	CM
M1/pro-inflammatory macrophage markers	CD86	=	+1.86	+2	=	=	=
	TLR4	−10	−10	−10	−10	−10	−10
	iNOS	=	=	=	+1.5	=	=
	NF-kB-p65	=	=	=	=	=	=
	TNFα	+36	+20	+20	+40	+25	+20
	IL-8	+4	+4	+7	+12	+4	=
	IL-1β	+17	+10	+ 35	+7	+7	+30
	IFNα	=	=	=	=	=	=
	IL-6	+37	+10	+13	+12	+50	+21
M2/anti-inflammatory macrophage markers	CD163	+4.4	+3.2	+4	+2.6	+2.2	+2.3
	STAT6	=	=	+ 1.5	=	=	+1.8
	IL-10	+10	+50	+15	+23	+20	+12
	CCL17	=	=	=	+5	+5	=
	IL-4	+7	+7	+9	+3.5	+4.5	+13

Fold numbers of increase (+), decrease (−), or not modified (=) compared to M0 macrophages. The macrophage markers are categorized as M1 and M2. Inducible nitric oxide synthase (iNOS); cluster of differentiation 86 (CD86); major histocompatibility complex II (MHCII); cluster of differentiation 163 (CD163); signal transducer and activator of transcription 6 (STAT6); toll-like receptor 4 (TLR4); subunit p65 of nuclear factor kappa-light-chain-enhancer of activated B cells (NFkB-p65); interleukin 1β (IL-1β); interleukin 6 (IL-6); interferon α (IFN α); tumor necrosis factor α (TNFα); interleukin 10 (IL-10); C-C motif chemokine ligand 17 (CCL17). CM = conditioned medium of U87 MG cells. TMZ = temozolomide.

## Supplementary Materials

The following are available online at https://www.mdpi.com/1422-0067/21/21/8353/s1.
