# Supplementary material for: Molecular Characterization of Temozolomide-Treated and Non Temozolomide-Treated Glioblastoma Cells Released Extracellular Vesicles and Their Role in the Macrophage Response
Source: Int J Mol Sci. 2020 Nov 7;21(21):8353. doi: 10.3390/ijms21218353 (PMC7664451; doi:10.3390/ijms21218353)
Supplement: Supplementary file 1 [file ijms-21-08353-s001.pdf]

Supplementary Material

Supplementary Methods

MTT assay

To determine the concentration of TMZ, cells were seeded at a density of 15 × 10^4/well in a 6-well plate for 24h to allow the attachment to substrate, and treated with TMZ (50, 100, 200 and 400 μM) or vehicle for 24, 48 and 72h. The culture medium was discharged, the cells were washed two times with Phosphate-Buffered Saline (PBS) 0,2 M pH 7,4 and fresh culture medium containing 1 mg/mL of 3-(4,5-dimethylthiazol-2-yl)-2,5-diphenyltetrazolium bromide salt (MTT) was added to each well. After 2h of incubation at 37 °C in a 5% CO_2 humidified atmosphere, MTT was reduced to formazan salt, a dark insoluble product, by the mitochondrial reductase of vital cells. Then, formazan salts were dissolved in dimethylsulfoxide (DMSO), leading to a violet solution whose absorbance was measured with Ultrospec 4000 UV–visible spectrophotometer (Pharmacia Biotech, Stockholm, Sweden) at 570 nm. Viability was expressed as percentage of the Relative Growth Rate (RGR) by the equation:

$$RGR = (D_{sample}/D_{control})/100$$

where D_{sample} and $D_{control}$ are the absorbance of the test samples and the negative controls, respectively.

Supplementary Figure 1

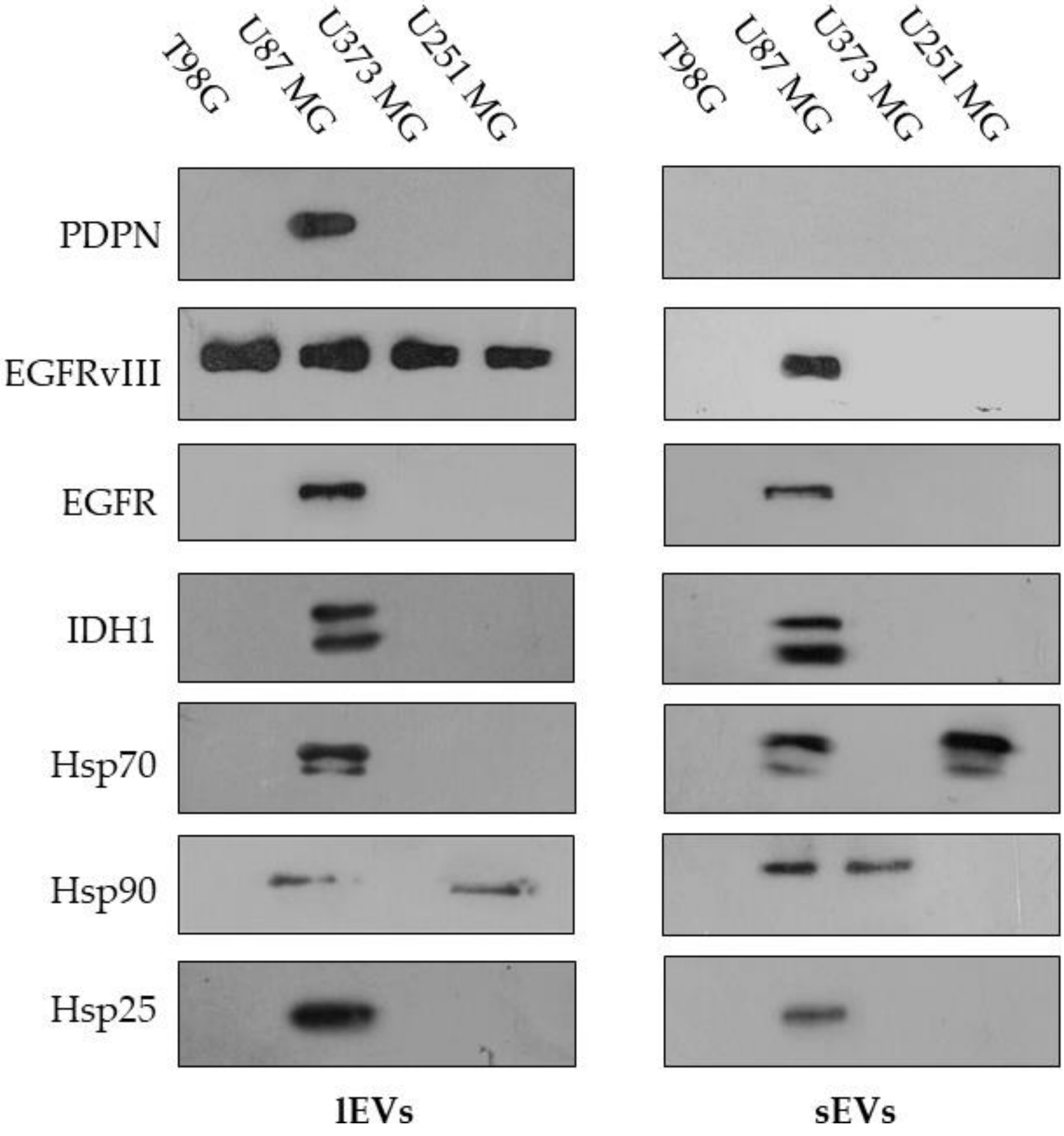

Western Blot analysis of markers in large and small EVs isolated from T98 G, U-87 MG, U-373 MG and U-251 MG cells. Analysis was performed as reported in Materials and Methods. 10 μg of proteins were loaded for each cell line for gel electrophoresis.

EGFR: epidermal growth factor receptor, EGFRvIII: epidermal growth factor receptor variant III, IDH1: isocitrate dehydrogenase 1, PDPN: podoplanin, Hsp25: Heat shock protein 25, Hsp70: Heat shock protein 70; Hsp90: Heat shock protein 90.

Supplementary Figure 2

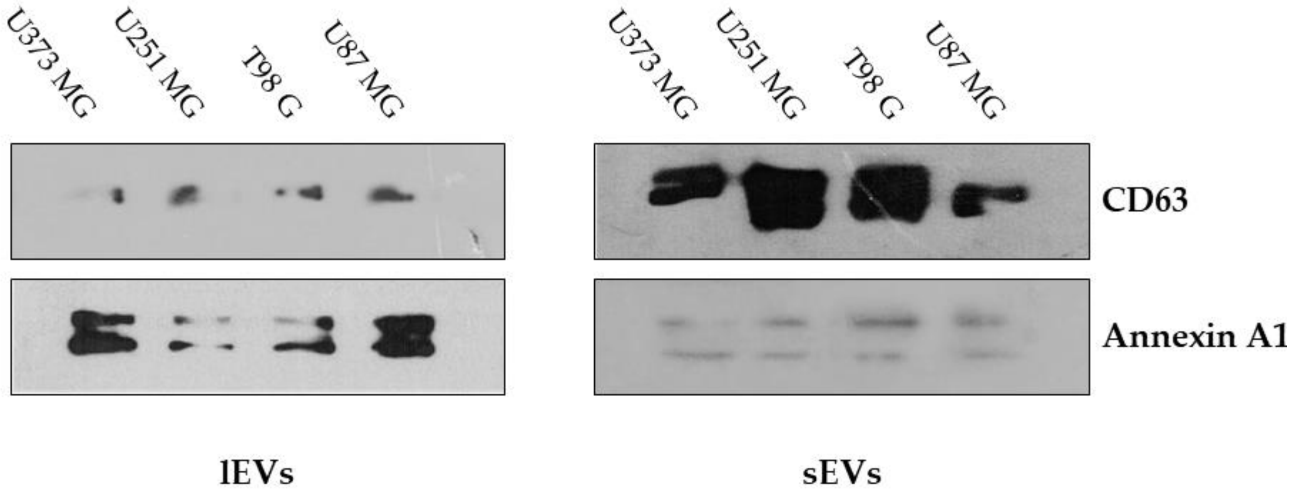

Western Blot analysis of specific markers of small (CD63) and large (Annexin A1) vesicles isolated from U373 MG, U251 MG, T98 G and U87 MG cells. Analysis was performed as reported in Materials and Methods. 10 μg of proteins were loaded for each cell line for gel electrophoresis.

Supplementary Figure 3.

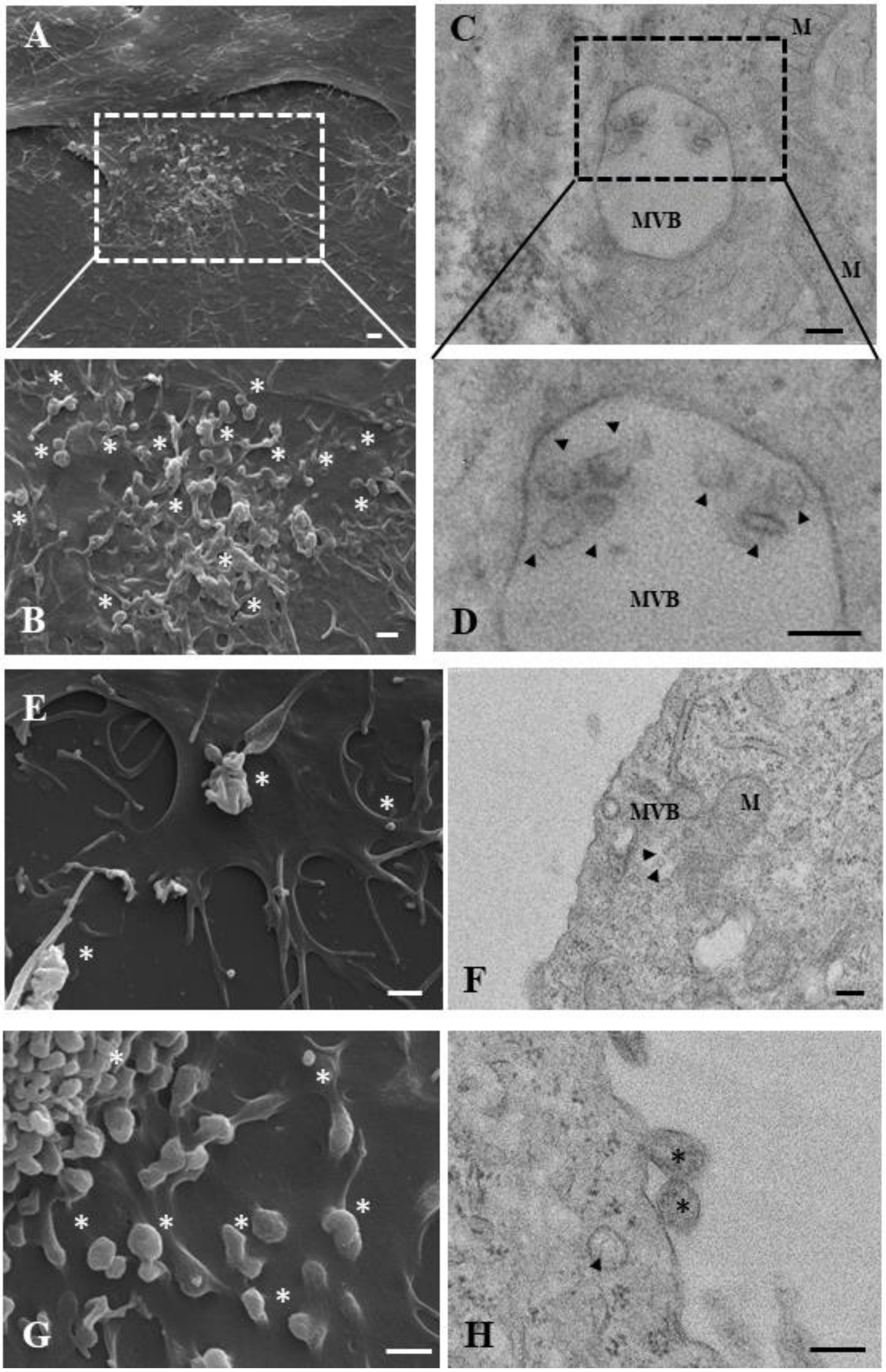

Transmission (A, B, G, E) and Scanning (C, D, H, F) electron micrographs of U-251 MG (A-D), T98 G (E, F) and U-373 MG (G, H) cells. Micrographs show large EVs (asterisks) shed from plasma membrane and small EVs (arrow heads) inside multivesicular body (MVB) (dashed black line); M: mitochondria. Bar = 200 nm

Supplementary Figure 4.

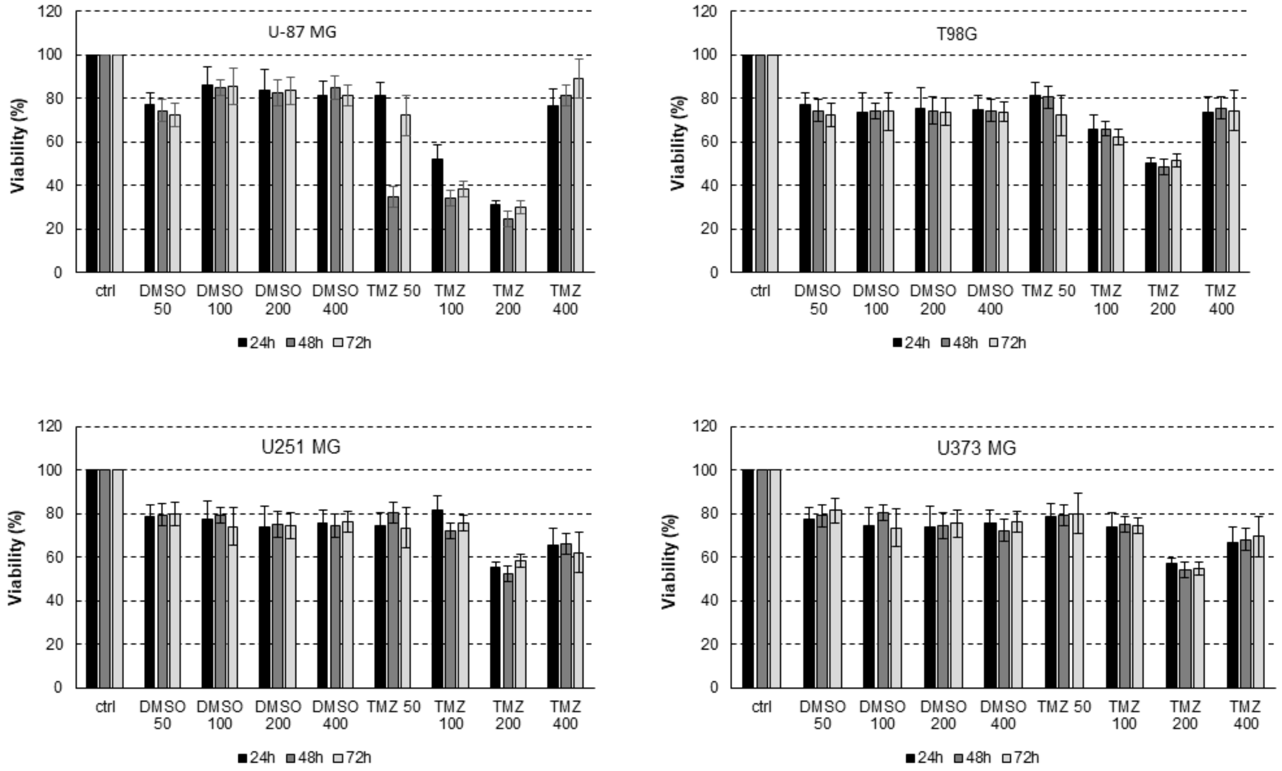

MTT cell viability. The percentages of viable GBM cells following treatment with different Temozolomide concentration (50, 100, 200, 400 μM) for 24, 48 and 72 hours are reported. All values are given as percentage referred to control values, taken as 100%. Each error bar represents the SE of three independent experiments, performed in triplicate. All values are significant ($p<0.05$) with respect to untreated cells. Abbreviations: Ctrl = untreated cells at 0 hours, TMZ = temozolomide, DMSO = control of toxicity of vehicle of TMZ

Supplementary Figure 5.

A

	EGFR		EFGRvIII		IDH1		PDPN		Hsp25		Hsp70		Hsp90	
	- TMZ	+ TMZ	- TMZ	+ TMZ	- TMZ	+ TMZ	- TMZ	+ TMZ	- TMZ	+ TMZ	- TMZ	+ TMZ	- TMZ	+ TMZ
cells	2,476258 ±0,067	1,256587 ±0,013*	1,359621 ±0,098	0,689541 ±0,033*	2,514781 ±0,054	2,498521 ±0,077	2,615874 ±0,071	1,296698 ±0,063*	2,598854 ±0,071	2,586638 ±0,058	3,356214 ±0,072	1,715521 ±0,053*	1,265568 ±0,093	1,261106 ±0,041
lVEs	2,251486 ± 0,073	0,685455 ±0,0098*°	0,736514 ±0,057*	0,624781 ±0,031*°	4,95862 ±0,066*	3,748999 ±0,088*°	7,847622 ±0,085*	1,328547 ±0,091*°	1,275541 ±0,098*	0,637135 ±0,038*°	15,01254 ±0,087*	3,395481 ±0,039°	1,259964 ±0,013	1,272354 ±0,031
sVEs	0,552221 ± 0,029*#	0,695471 ±0,063*°	0,652471 ±0,064*#	0,645874 ±0,088*	6,286944 ±0,071*#	3,785477 ±0,067*°	N.D.	N.D.	2,600541 ±0,068#	2,596633 ±0,081#	13,54872 ±0,081*	3,381489 ±0,081°	2,615474 ±0,055*#	2,086351 ±0,041*#°

Densitometric analysis of western blots of epidermal growth factor receptor (EGFR), EGFRvIII, isocitrate dehydrogenase 1 (IDH1), podoplanin (PDPN), Hsp25, Hsp70 and Hsp90 in U-87 MG cells and in isolated lEVs and sEVs after 200 μM temozolomide (TMZ) for 48 hours treatment. The values for all experimental groups reported represent the mean ± SD (n = 3) of three independent experiments; (∗) p < 0,05 compared to respective origin cells; (°) p < 0,05 significance between (–TMZ) and (+TMZ) values; (#) p < 0,05 significance between sEVs and lEVs values.

B

	CD86		CD163		iNOS		STAT6	
	- TMZ	+ TMZ	- TMZ	+ TMZ	- TMZ	+ TMZ	- TMZ	+ TMZ
lVEs	0,913013 ±0,025	1,348677 ±0,014°	2,476258 ±0,063	1,375942 ±0,051°	1,253695 ± 0,013	1,356282 ± 0,036°	0,752221 ± 0,029	1,469487 ± 0,083°
sVEs	1,814154 ±0,031#	1,350960 ±0,009°	1,762284 ±0,025#	1,187442 ± 0,045°	1,091543 ± 0,014#	1,225104 ± 0,082#°	0,886214 ± 0,037#	1,452368 ±0,043°
CM	1,781783 ±0,018#	1,379730 ±0,015°	2,251486 ± 0,078§	1,25672 ± 0,029°§	1,274837 ± 0,014§	1,120497 ± 0,004#°§	1,635289 ± 0,065#§	1,797753 ± 0,072#°§

Densitometric analysis of western blots of CD86, CD163, iNOS and STAT6 in M0 macrophages after exposure to 200 μM temozolomide (TMZ) for 48 hours treated or non-TMZ- treated U-87 MG cells derived lEVs, sEVs and free EVs CM for 24 hours. The values for all experimental groups reported represent the mean ± SD (n = 3) of three independent experiments; (§) p < 0,05 significance between sEVs and CM values; (°) p < 0,05 significance between (–TMZ) and (+TMZ) values; (#) p < 0,05 significance between lEVs, sEVs and CM values.

Supplementary Figure 6.

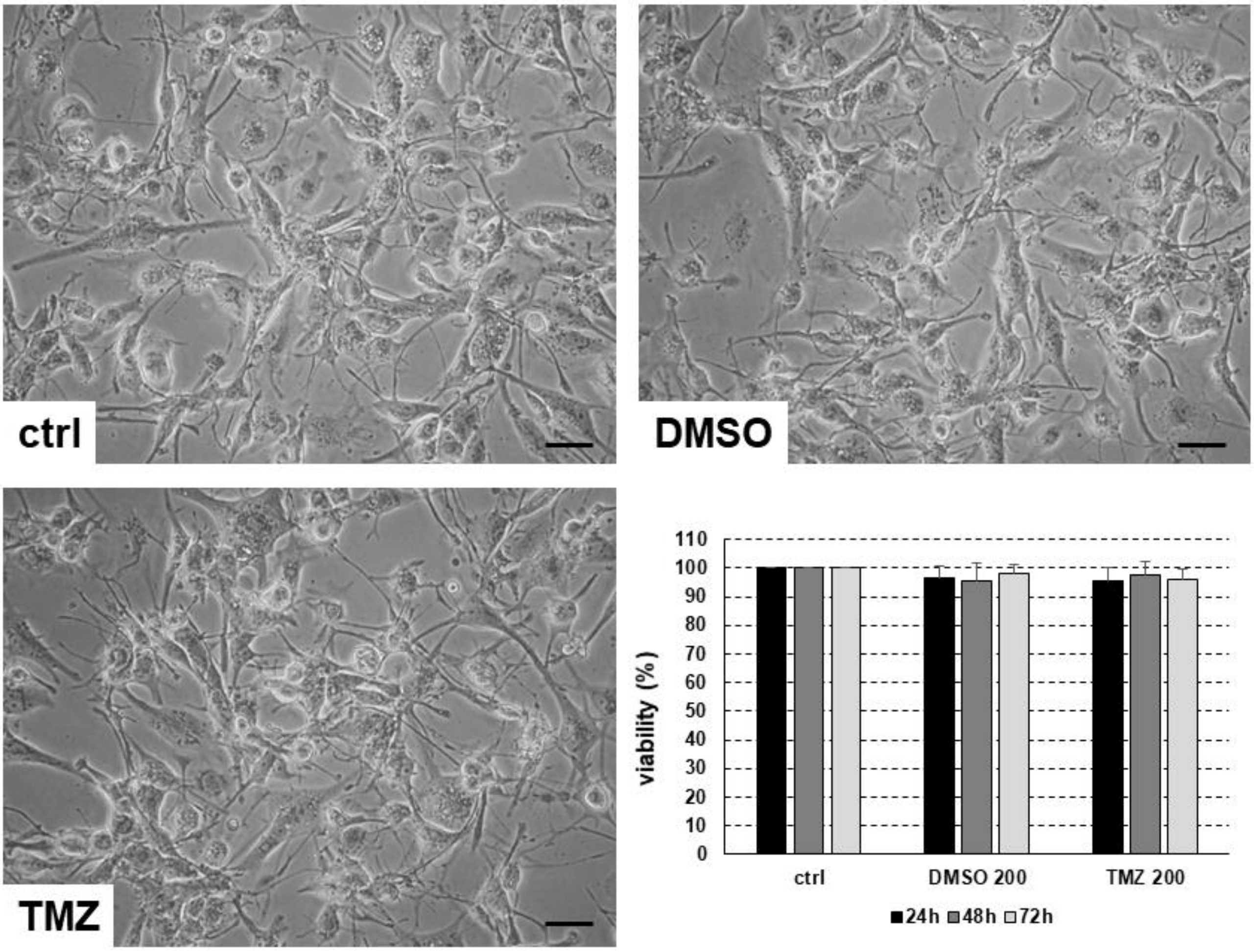

Representative light microscopy micrographs of M0 macrophages (ctrl), DMSO-treated or TMZ-treated M0 macrophages. Bar = 10μm

MTT cell viability. The percentages of viable GBM cells following treatment with 200 μM Temozolomide (TMZ) for 24, 48 and 72 hours are reported. All values are referred to control values at 0 hour, taken as 100%. Each error bar represents the SE of three independent experiments, performed in triplicate.

Abbreviations: Ctrl = untreated M0 at 0 hour, TMZ 200 = 200 μM Temozolomide, DMSO 200 = control of toxicity of TMZ vehicle solution.

Supplementary Figure 7

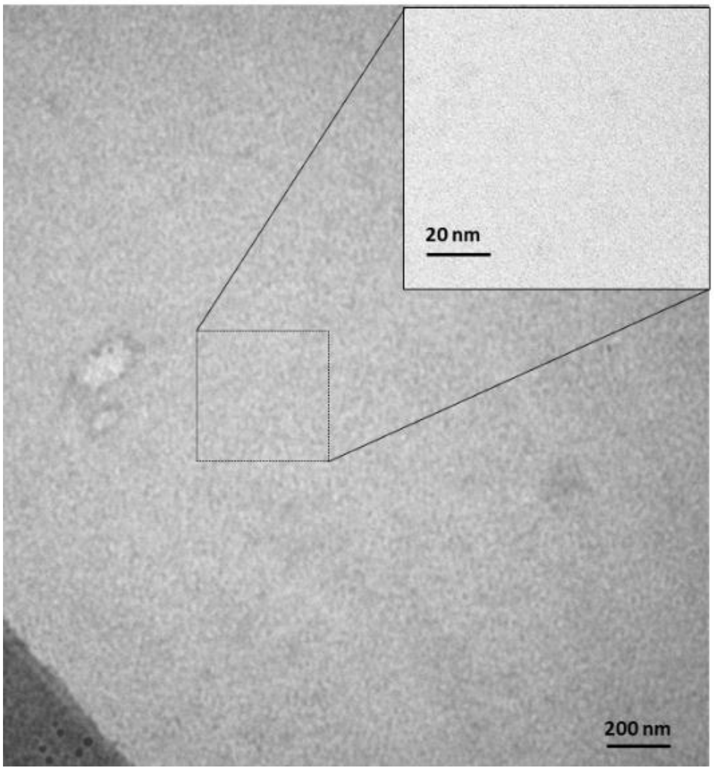

Transmission electron (TEM) micrograph of EVs free conditioned medium. The culture medium has been observed after over-night centrifugation at 100,000g at 4°C to remove EVs.
